# Targeting transforming growth factor β to enhance cancer immunotherapy

**DOI:** 10.3390/curroncol13040015

**Published:** 2006-08

**Authors:** T. Hahn, E.T. Akporiaye

**Keywords:** dc-based vaccine, tgfβ, immunotherapy of cancer

## 1. INTRODUCTION

Human tumours have evolved intricate mechanisms to evade the immune system, either by avoiding recognition or by inhibiting and eliminating immune cells. Although clear evidence exists that the immune system is mobilized in response to tumour growth—a fact that is manifested in the accumulation of numerous immune cells including dendritic cells (dcs), natural killer (nk) cells, macrophages, and lymphocytes at the tumour site—tumour growth continues unabated. One of the major obstacles to the success of immunotherapy of cancer is therefore the diminished immune responsiveness of cancer patients with progressing disease.

Continued tumour growth in the presence of what appears to be a significant antitumour response suggests the existence of a microenvironment that is inhospitable to antitumour immune effector mechanisms. Several tumour-derived products present in sera and tumour extracts from cancer patients, including transforming growth factor β (tgfβ), prostaglandin E2, interleukin-10, and vascular endothelial growth factor 1–4, have been identified and employ a variety of mechanisms to promote tumour growth.

To develop effective cancer therapy modalities, an understanding of the biologic effects that these tumour-derived factors have on immune cells is essential. The challenge, therefore, is to identify the underlying mechanisms of action of tumour-promoting factors, so that effective counteractive strategies that will bolster the antitumour immune response can be developed.

## 2. DISCUSSION

### 2.1 TGFβ and Dendritic Cells

One of the most studied and best described tumour-derived factors with potent immunosuppressive activity is tgfβ. A pleiotropic cytokine 5, tgfβ is secreted in copious amounts by murine and human cancer cells. In melanoma and in pancreatic, colorectal, lung, and breast cancers, elevated levels of circulating tgfβ have been correlated with disease progression, metastases, disease recurrence, and mortality 6–13. The immunosuppressive activities of tgfβ include inhibition of the differentiation and effector functions of T cells, nk cells, lymphokine-activated killer cells, macrophages, and dcs 14.

The impact of tumour-derived tgfβ on dc function is of particular interest because of the foundational role that dcs play in the induction of antigen-specific tumour immunity—especially in the context of their use as cancer vaccines. Transforming growth factor β affects dcs in numerous ways that negatively affect their ability to prime tumour-specific T cells, including downregulation of cell-surface major histocompatibility complex (mhc) antigens, downregulation of co-stimulatory molecules and chemokine receptors, and impairment of maturation and migration to secondary lymphoid organs. These effects are also supported by the reported functional impairment of circulating and tumour-infiltrating dcs in tumour-bearing animals and in cancer patients such that the dcs are unable to induce T-cell responses 15–18.

Given the central role of dcs in inducing antigen-specific immune responses, the impact of tumour-derived tgfβ on dcs poses a major challenge for dc-based cancer immunotherapy. Therapeutic strategies that can successfully negate the impact of tgfβ on dcs would therefore be expected to result in enhanced dc function and to lead to improved clinical responses.

The importance of dc-mediated immune responses against tgfβ-producing tumours can be shown by systemic inhibition of tgfβ-mediated signalling using a tgfβ neutralizing antibody in combination with a dc vaccine or dcs in which tgfβ signalling is impaired by the expression of a dominant negative tgfβ type ii receptor (dntgfβrii) transgene 15,19 (unpubl. data). These strategies have enhanced the immune response against tgfβ-producing murine breast cancer and melanoma respectively. Those results suggest that counteracting tgfβ-mediated signalling in immune cells, including dcs, may be exploited to enhance antitumour immune responses. However, tumour cell destruction by the immune system ultimately depends on an efficient cell-mediated immune response carried out by cd8+ cytotoxic T lymphocytes (ctls). This process usually depends on mhc class-ii restricted cd4+ T-cell help that provides the cytokine signals necessary for activation and clonal expansion of tumour reactive ctls and for memory formation that can then provide the much-desired long-term antitumour protection.

Like dcs, ctls and cd4+ helper T cells are both functionally impaired in the presence of tgfβ, as evidenced by reduced cytokine production and proliferative potential, blunting the effectiveness of the cell-mediated immune response. The importance of this process and the impact of neutralizing the immunosuppressive effects of TGFβ on T cells to enhance an antitumour immune response *in vivo* has been demonstrated by Flavell’s group. Their studies 19,20 targeted expression of dntgfβrii in transgenic mice to T lymphocytes, leading to immune-mediated rejection of tgfβ-producing el-4 thymoma and B16 melanoma tumours. In a study by another group, sub-lethally irradiated mice transplanted with dntgfβrii-expressing bone marrow cells demonstrated improved survival over mice receiving wild-type bone marrow when challenged with B16 melanoma or tramp-C2 prostate tumours 21. Furthermore, adoptive transfer of tumour-specific, dntgfβrii-expressing cd8+ T cells into tramp-C2 tumour-bearing mice led to improved survival and a reduction in the number of pulmonary metastases 22.

### 2.2 Novel Human Therapies

Promising findings of augmentation of the antitumour immune response by inhibition of tgfβ leads us to examine how such augmentation may be translated into novel human therapies. Although the described animal models are useful for studying *in vivo* dc, T-cell, and tgfβ biology, they do not easily lend themselves to translation to the clinic for the treatment of human malignancies. One approach would be to generate tumour-specific T cells in combination with antigen-pulsed dcs for adoptive cell transfer (act).

Current act involves the transfer of either *ex vivo* activated T cells or antigen-pulsed dcs into cancer patients. Adoptive transfer of tumour-specific T cells has been accomplished by first vaccinating patients with autologous tumour cells, harvesting the vaccine-draining lymph node cells, expanding the recovered lymphocytes *ex vivo,* and re-infusing the activated T cells into the patient 23,24. In similar studies, treatment with *ex vivo–*stimulated tumour-infiltrating T lymphocytes was well tolerated, and the transferred T cells persisted in the circulation for several months 25. The dc-based act has used tumour lysate, tumour antigen, or peptide-pulsed dcs, and has demonstrated robust immunologic antitumour responses accompanied by modest clinical outcomes in patients with cancers of various histologic origins 26–29.

These adoptively transferred dcs and T cells would be expected to encounter significant amounts of tumour-derived tgfβ in the peripheral circulation and at the tumour site that could impair their ability to efficiently prime the immune system or mediate effector functions. A combinatorial approach in which both dcs and T cells are protected from the deleterious effects of tgfβ would be expected to improve the therapeutic efficacy of act. Several different classes of tgfβ antagonists are now in development, such as tgfβ-neutralizing monoclonal antibodies 30,31, soluble tβrii:fc fusion proteins, and antisense tgfβ oligonucleotides 32,33.

Of particular interest is the recent development of small-molecule tgfβ receptor kinase inhibitors. These molecules resemble dntgfβrii expression in that they inhibit tgfβ-mediated signalling at the cellular level 34–36. They are particularly promising because of their ease of manufacture and use as compared with larger-molecular therapeutics such as antibodies.

## 3. CONCLUSION

The observations presented here show that therapeutic approaches that neutralize or abrogate tumour-derived products such as tgfβ significantly enhance the effector activity of immunocytes in the tumour microenvironment. Studies are underway to combine these novel anti-tgfβ therapies with adoptive cell transfer to devise better therapeutic approaches that, in conjunction with established treatments, will provide oncologists with additional therapeutic options in the future.

## References

[1] Sulitzeanu D (1993). Immunosuppressive factors in human cancer. Adv Cancer Res.

[2] Dix AR, Brooks WH, Roszman TL, Morford LA (1999). Immune defects observed in patients with primary malignant brain tumors. J Neuroimmunol.

[3] Marincola FM, Jaffee EM, Hicklin DJ, Ferrone S (2000). Escape of human solid tumors from T-cell recognition: molecular mechanisms and functional significance. Adv Immunol.

[4] Rosenberg SA, Yang JC, Restifo NP (2004). Cancer immunotherapy: moving beyond current vaccines. Nat Med.

[5] Massague J (1998). TGF-β signal transduction. Annu Rev Biochem.

[6] Fynan TM, Reiss M (1993). Resistance to inhibition of cell growth by transforming growth factor-β and its role in oncogenesis. Crit Rev Oncog.

[7] Bellone G, Turletti A, Artusio E (1999). Tumor-associated transforming growth factor-β and interleukin-10 contribute to a systemic Th2 immune phenotype in pancreatic carcinoma patients. Am J Pathol.

[8] Shim KS, Kim KH, Han WS, Park EB (1999). Elevated serum levels of transforming growth factor-β1 in patients with colorectal carcinoma: its association with tumor progression and its significant decrease after curative surgical resection. Cancer.

[9] Krasagakis K, Tholke D, Farthmann B, Eberle J, Mansmann U, Orfanos CE (1998). Elevated plasma levels of transforming growth factor (tgf)-β1 and tgf-β2 in patients with disseminated malignant melanoma. Br J Cancer.

[10] Kong FM, Anscher MS, Murase T, Abbott BD, Iglehart JD, Jirtle RL (1995). Elevated plasma transforming growth factor-β 1 levels in breast cancer patients decrease after surgical removal of the tumor. Ann Surg.

[11] Tsushima H, Kawata S, Tamura S (1996). High levels of transforming growth factor β 1 in patients with colorectal cancer: association with disease progression. Gastroenterology.

[12] Gorsch SM, Memoli VA, Stukel TA, Gold LI, Arrick BA (1992). Immunohistochemical staining for transforming growth factor β 1 associates with disease progression in human breast cancer. Cancer Res.

[13] Walker RA, Dearing SJ (1992). Transforming growth factor β 1 in ductal carcinoma *in situ* and invasive carcinomas of the breast. Eur J Cancer.

[14] de Visser KE, Kast WM (1999). Effects of tgf-β on the immune system: implications for cancer immunotherapy. Leukemia.

[15] Kobie JJ, Wu RS, Kurt RA (2003). Transforming growth factor β inhibits the antigen-presenting functions and antitumor activity of dendritic cell vaccines. Cancer Res.

[16] Gabrilovich DI, Ciernik IF, Carbone DP (1996). Dendritic cells in antitumor immune responses. I. Defective antigen presentation in tumor-bearing hosts. Cell Immunol.

[17] Gabrilovich DI, Nadaf S, Corak J, Berzofsky JA, Carbone DP (1996). Dendritic cells in antitumor immune responses. II. Dendritic cells grown from bone marrow precursors, but not mature dc from tumor-bearing mice, are effective antigen carriers in the therapy of established tumors. Cell Immunol.

[18] Gabrilovich DI, Corak J, Ciernik IF, Kavanaugh D, Carbone DP (1997). Decreased antigen presentation by dendritic cells in patients with breast cancer. Clin Cancer Res.

[19] Gorelik L, Flavell RA (2000). Abrogation of tgfβ signaling in T cells leads to spontaneous T cell differentiation and autoimmune disease. Immunity.

[20] Gorelik L, Flavell RA (2001). Immune-mediated eradication of tumors through the blockade of transforming growth factor-β signaling in T cells. Nat Med.

[21] Shah AH, Tabayoyong WB, Kundu SD (2002). Suppression of tumor metastasis by blockade of transforming growth factor β signaling in bone marrow cells through a retroviral-mediated gene therapy in mice. Cancer Res.

[22] Zhang Q, Yang X, Pins M (2005). Adoptive transfer of tumor-reactive transforming growth factor-β-insensitive cd8+ T cells: eradication of autologous mouse prostate cancer. Cancer Res.

[23] Meijer SL, Dols A, Urba WJ (2002). Adoptive cellular therapy with tumor vaccine draining lymph node lymphocytes after vaccination with hla-B7/β_2_-microglobulin gene-modified autologous tumor cells. J Immunother.

[24] Chang AE, Li Q, Jiang G, Sayre DM, Braun TM, Redman BG (2003). Phase ii trial of autologous tumor vaccination, anti-cd3-activated vaccine-primed lymphocytes, and interleukin-2 in stage iv renal cell cancer. J Clin Oncol.

[25] Dudley ME, Wunderlich JR, Robbins PF (2002). Cancer regression and autoimmunity in patients after clonal repopulation with antitumor lymphocytes. Science.

[26] Banchereau J, Palucka AK (2005). Dendritic cells as therapeutic vaccines against cancer. Nat Rev Immunol.

[27] Geiger JD, Hutchinson RJ, Hohenkirk LF (2001). Vaccination of pediatric solid tumor patients with tumor lysate-pulsed dendritic cells can expand specific T cells and mediate tumor regression. Cancer Res.

[28] Lau R, Wang F, Jeffery G (2001). Phase i trial of intravenous peptide-pulsed dendritic cells in patients with metastatic melanoma. J Immunother.

[29] Yu JS, Liu G, Ying H, Yong WH, Black KL, Wheeler CJ (2004). Vaccination with tumor lysate-pulsed dendritic cells elicits antigen-specific, cytotoxic T-cells in patients with malignant glioma. Cancer Res.

[30] Arteaga CL, Hurd SD, Winnier AR, Johnson MD, Fendly BM, Forbes JT (1993). Anti-transforming growth factor (tgf)-β antibodies inhibit breast cancer cell tumorigenicity and increase mouse spleen natural killer cell activity. Implications for a possible role of tumor cell/host tgf-β interactions in human breast cancer progression. J Clin Invest.

[31] Wojtowicz–Praga S, Verma UN, Wakefield L, Esteban JM, Hartmann D, Mazumder A (1996). Modulation of B16 melanoma growth and metastasis by anti-transforming growth factor β antibody and interleukin-2. J Immunother Emphasis Tumor Immunol.

[32] Yang YA, Dukhanina O, Tang B (2002). Lifetime exposure to a soluble tgf-β antagonist protects mice against metastasis without adverse side effects. J Clin Invest.

[33] Muraoka RS, Dumont N, Ritter CA (2002). Blockade of tgf-β inhibits mammary tumor cell viability, migration, and metastases. J Clin Invest.

[34] Uhl M, Aulwurm S, Wischhusen J, Weiler M (2004). sd-208, a novel transforming growth factor β receptor i kinase inhibitor, inhibits growth and invasiveness and enhances immunogenicity of murine and human glioma cells *in vitro* and *in vivo.*. Cancer Res.

[35] Singh J, Chuaqui CE, Boriack–Sjodin PA (2003). Successful shape-based virtual screening: the discovery of a potent inhibitor of the type i tgfβ receptor kinase (tβri). Bioorg Med Chem Lett.

[36] Peng SB, Yan L, Xia X (2005). Kinetic characterization of novel pyrazole tgf-β receptor i kinase inhibitors and their blockade of the epithelial–mesenchymal transition. Biochemistry.

